# DNA Methylation and Asthma Acquisition during Adolescence and Post-Adolescence, an Epigenome-Wide Longitudinal Study

**DOI:** 10.3390/jpm12020202

**Published:** 2022-02-02

**Authors:** Aniruddha Rathod, Hongmei Zhang, Syed Hasan Arshad, Susan Ewart, Caroline L. Relton, Wilfried Karmaus, John W. Holloway

**Affiliations:** 1 Division of Epidemiology, Biostatistics and Environmental Health, School of Public Health, University of Memphis, Memphis, TN 38111, USA; abrathod@memphis.edu (A.R.); karmaus1@memphis.edu (W.K.); 2 Clinical and Experimental Sciences, Faculty of Medicine, University of Southampton, Southampton SO16 6YD, UK; S.H.Arshad@soton.ac.uk; 3 The David Hide Asthma and Allergy Research Centre, St Mary’s Hospital, Newport, Isle of Wight PO30 5TG, UK; 4 NIHR Southampton Biomedical Research Centre, University Hospital Southampton, Southampton SO16 6YD, UK; J.W.Holloway@soton.ac.uk; 5 College of Veterinary Medicine, Michigan State University, East Lansing, MI 48824, USA; ewarts@msu.edu; 6 MRC Integrative Epidemiology Unit, University of Bristol, Bristol BS8 1QU, UK; caroline.relton@bristol.ac.uk; 7 Population Health Sciences, Bristol Medical School, University of Bristol, Bristol BS8 1QU, UK; 8 National Institute for Health Research Bristol Biomedical Research Centre, University Hospitals Bristol and Weston NHS Foundation Trust, University of Bristol, Bristol BS8 2BN, UK; 9 Human Development and Health, Faculty of Medicine, University of Southampton, Southampton SO16 6YD, UK

**Keywords:** DNA methylation, IOWBC, ALSPAC, asthma transition, sex-specificity

## Abstract

The role of epigenetics in the pathogenesis of asthma acquisition in adolescence and post-adolescence has been unknown. We carried out a longitudinal epigenome-wide association study, using data from the Isle of Wight Birth Cohort (IOWBC). To improve statistical power, we first screened CpGs based on associations of DNA methylation (DNAm) at an age of 10 years (pre-adolescence) with asthma acquisition at 10–18 years (during adolescence). A logistic regression with repeated measures was applied to CpGs that passed screening to examine the associations of pre-adolescence DNAm with asthma acquisition from 10–18 years and 18–26 years, with an interaction term to evaluate transition period specificity. Findings were further tested in an independent birth cohort, ALSPAC. In total, 205 CpGs (with 150 being females) showed associations with asthma acquisition (main or interaction effects) at FDR = 0.05 in IOWBC, of which 112 (90 being females) showed consistent associations in the ALSPAC. Genes that the identified CpGs were mapped to, e.g., *AKAP1* and *ENO1*, have been shown to be associated with the risk of asthma. Our findings indicated that DNAm at specific CpGs was associated with asthma acquisition. CpGs showing such associations were likely to be different between males and females and, at certain CpGs, were unique to a specific transition period.

## 1. Introduction

Asthma is the most prevalent chronic respiratory condition [1], affecting 1–18% of the population in several countries [2]. Over recent decades, childhood asthma has become a major public health issue [3], with an increasing prevalence worldwide [4]. Environmental factors such as air pollution, infectious agents, and tobacco smoke have been shown to be associated with the development of asthma [5].

DNA methylation (DNAm), a robust and stable epigenetic mark, represents a potential mechanism for environmental impact in human diseases [6]. Recent studies suggest that DNAm signatures of cytosine-phosphate-guanines (CpG) sites are associated with asthma [7,8,9]. Since peripheral blood is readily obtainable and easy to handle in laboratory processing, and information of immune cells in blood is relevant to asthma pathogenesis [10], DNAm in peripheral blood cells has been commonly examined in epigenome-wide studies of asthma [11,12,13,14].

While the development of asthma clearly reflects the combination of inherited susceptibility and environmental exposures, the pathogenesis and underlying biological mechanisms involved in the onset of asthma later in life are not well understood. Asthma most commonly develops during early childhood [15], and the prevalence of asthma depends on gender and age. Asthma is more prevalent among pre-adolescent boys, while it becomes more prevalent among females after puberty, with prevalence in males and females being approximately equal in adulthood [16,17,18]. However, the pathogenesis of these sex differences in asthma across adolescence and adulthood remain unclear.

Although previous studies have demonstrated an association between DNAm and asthma, the role that DNAm plays in asthma acquisition, especially during the critical transition period from pre- to post-adolescence, and how its role changes over time, e.g., from adolescence to adulthood, are unknown. Findings from this type of study will not only identify important markers for asthma acquisition but, more importantly, will also benefit our future efforts in asthma prediction and, consequently, asthma prevention. To this end, in this study, for each gender, we examined the association between DNAm at pre-adolescence and asthma acquisition from pre- to post-adolescence (10 to 18 years) and between DNAm at post-adolescence and asthma acquisition from post-adolescence to adulthood (18–26 years), utilizing genome-wide DNA methylation data. We hypothesized that DNAm at specific CpG sites measured before disease onset, either in pre- or post-adolescence, would be associated with asthma acquisition both during adolescence and in later adulthood and that there would be differences in such DNA methylation patterns by time window (adolescence or post-adolescence) and by gender. Furthermore, the identified CpGs were used to identify biological processes to better understand asthma pathogenesis, which will further promote the identification of potential preventive and/or therapeutic targets.

## 2. Methods

### 2.1. Study Population

The study population comprised of children born between 1 January 1989 and 28 February 28 1990 on the Isle of Wight (IoW), UK (IOWBC) [19]. Out of the 1536 children born and recruited, 1456 in the IOWBC were available for further follow-up at ages 1, 2, 4, 10, 18, and 26 years. Ethics approval was obtained by the local research ethics committee (NRES Committee South Central—Hampshire B) (06/Q1701/34) [20].

### 2.2. Asthma Acquisition

Questionnaires that included the questions of the International Study of Asthma and Allergy in Childhood (ISAAC) were completed by parents/participants at ages 4, 10, 18, and 26 years [8,21,22,23,24]. Asthma was defined as “physician-diagnosed asthma” and “wheezing or whistling in the chest in the last 12 months” or “current treatment for asthma.” Subjects with asthma at an age of 4 years were excluded. The outcome used in this study, asthma acquisition, was defined as individuals who were asthma free at an age of 10 years and recorded as having asthma at an age of 18 years (no→yes). The same definition was applied for asthma acquisition from 18 to 26 years (no→yes). Subjects who did not have asthma at both the transition periods were taken as a reference (no→no).

### 2.3. DNA Methylation

Using a standardized salting procedure, DNA was extracted from peripheral whole blood samples collected at ages 10 and 18 years [25]. Fluorometric quantitation was used to estimate DNA concentration. Methylation levels at each CpG site were measured using Illumina Infinium HumanMethylation450 or MethylationEPIC BeadChips (Illumina, Inc., San Diego, CA, USA). Probes that did not reach a *p*-value of 10^−16^ in at least 95% of samples were excluded. The same criterion was applied to exclude samples, i.e., samples with a *p*-value >10^−16^ in at least 95% of the CpGs. CpGs on the sex chromosome were excluded.

Using a CPACOR pipeline, DNA methylation (DNAm) was pre-processed for the data from both HumanMethylation450 and MethylationEPIC. DNAm intensities were quantile normalized using the *minim* R computing package [26]. The quantile normalized intensities at autosomal probes were then converted to beta values [27]. Principal components (PCs) inferred based on control probes were used to represent latent chip-to-chip and technical variation. We determined PCs based on the DNAm at shared control probes of the two DNAm platforms, HumanMethylation450 and MethylationEPIC. In total, 195 shared control probes were used to calculate the control probe PCs, with the top 15 PCs being included in our study to represent latent batch factors [28]. In this study, CpG sites common between the Illumina 450k platform and EPIC platform were examined. In addition, CpG sites were excluded if the minor allele frequency of a probe SNP at that site was >0.7% (i.e., ~≥10 out of 1456 subjects were expected to have the minor allele in the cohort) and the probe SNP was within 10 base pairs of the targeted CpG site. After quality control and pre-processing, 442,475 CpG sites were included in subsequent analysis.

Since whole blood is a mixture of distinct cell types [29], there was a need to adjust for cell-type composition to account for their potentially confounding effects [30]. Cell-type proportions were estimated using the Bioconductor *minfi* package [31,32]. The estimated cell-type proportions of CD4+ T cells, natural killer cells, neutrophils, B cells, monocytes, and eosinophil cells were included in the analyses as confounders.

### 2.4. Covariates

Atopic status was evaluated at ages 10 and 18 years based on results from a skin prick test (SPT) for 11 common allergens (house dust mite, cat dander, dog dander, grass pollen mix, tree pollen mix, *Alternaria alternate*, *Cladosporium herbarium*, cow’s milk, hen’s egg, peanuts, and cod). Being SPT positive to one or more of the 11 allergens was treated as being atopic. An active smoking status at 18 and 26 years was recorded as ‘yes’ if the participant was a current smoker at that respective age. Second-hand smoke exposure was coded at ages 18 and 26 years using information obtained from the smoking status of parents and other smokers in the household. To evaluate the contribution of the transition periods, 10–18 and 18–26 years, to the association of DNAm with asthma transition, transition periods were included in the analyses as adjusting factors.

### 2.5. Forced Exhaled Nitric Oxide (FeNO) and Lung Function Measurement

FeNO was measured at 18 and 26 years, according to the American Thoracic Society (ATS) guidelines. Lung function measurements, i.e., Forced Expiratory Volume in one second (FEV_1_) and Forced Vital Capacity (FVC), were measured using a KoKo Spirometer and software with a portable desktop device (both PDS instrumentation, Louisville, KY, USA), according to the ATS guidelines [33]. The FEV1/FVC ratio at 18 and 26 years were used for the analyses in this study [34].

### 2.6. Statistical Analyses

By regressing the M-values (base-2 logit transformed beta values of DNAm) at each CpG site on the aforementioned 15 PCs and 6 cell-type proportions, we obtained cell-type- and batch-adjusted DNAm (residuals) at each of the 442,475 CpG sites for each gender in the IOWBC. Screening of CpG sites was done to obtain DNAm potentially associated with asthma acquisition from pre- to post-adolescence using simple linear regressions. Here, asthma acquisition from 10 to 18 years of age was the independent variable, and DNAm at an age of 10 years was the dependent variable. The analysis was stratified by gender. For screening purposes, multiple testing was adjusted by controlling the false discovery rate (FDR) at a higher rate of 0.2. CpG sites that passed screening were included in subsequent analyses.

Logistic regressions with repeated measurements were applied to the CpGs that passed screening to evaluate the association of asthma acquisitions (no→yes) at two transition periods (10–18 years and 18–26 years) with DNAm at earlier ages (10 and 18 years, respectively). The use of repeated measures allowed us to achieve a higher statistical power and detect the desired effect size. This approach is especially desired in the situation of small sample sizes. Along with other covariates (atopic status at 10 and 18 years and an active and second-hand smoking status at 18 and 26 years), to assess whether the associations were different at different transition periods, in addition to the main effects of DNAm, we also tested interaction effects between DNAm and the transition period. For both situations (the models with main effects only and the models that included interaction effects), multiple testing was adjusted by controlling the FDR to be 0.05.

### 2.7. Replication Cohort: ALSPAC

In addition to the utilization of repeated measures in our statistical modelling, to further assess the informativity of the findings in the IOWBC, an independent replication cohort, the Avon Longitudinal study of Parents and Children (ALPSAC) cohort [35,36,37], was included to examine the CpGs showing significant interaction effects with transition periods in the IOWBC. DNAm in the ALSPAC cohort was assessed using the Infinium HumanMethylation450 BeadChip. DNAm data on 604 children in the ALSPAC cohort were available at ages 7 and 17 years [38]. DNAm pre-processing was performed by correcting for batch effects using the *minfi* package [26] and removing the CpGs with a detection *p*-value ≥0.01. Samples were flagged that contained a sex-mismatch based on X-chromosome methylation. Estimated cell-type proportions of CD4+ T cells, natural killer cells, neutrophils, B cells, monocytes, and granulocytes cells were used in the analyses to adjust for cell heterogeneity.

Asthma acquisition status from 7 to 17 years and 17 to 22 years was included in the analysis. It was defined as having no asthma at an age of 7 years and having asthma at an age of 17 years. The same definition was applied for asthma acquisition from 17 to 22 years. A logistic regression with repeated measurements was used with similar covariates (as those in the IOWBC) available in the ALSPAC, i.e., atopy status at an age of 7 years and second-hand smoke exposure status at ages 17 and 24 years. Please note that the study website contains details of all the data that are available through a fully searchable data dictionary and variable search tool (http://www.bristol.ac.uk/alspac/researchers/our-data/ (accessed on 12 August 2019)).

### 2.8. Association Analysis for FeNO and the FEV_1_/FVC Ratio

For CpGs showing a consistent association with asthma acquisition in both cohorts, they were further analysed for their longitudinal association with two objective markers for asthma severity, FeNO levels and the lung function parameter (FEV_1_/FVC ratio) [39,40]. The analysis was carried out for the two transition periods separately at these CpG sites using linear regressions, with DNAm being the independent variable and FeNO and the FEV_1_/FVC ratio being the dependent variables.

### 2.9. Detection of Differentially Methylated Regions (DMR)

Differentially methylated regions (DMRs) were identified using the *DMRcate* package in R [41]. To secure a sufficient number of CpGs for DMR enrichment analysis and to avoid missing important DMRs, the CpGs with a DNAm (in M values) associated with asthma acquisition via logistic regression at an FDR of 0.4 were included in the analysis.

### 2.10. Pathway Enrichment Analyses

For CpGs showing associations of DNAm with asthma acquisition status, the genes annotated to the CpGs were summarized along with information such as gene location and chromosome number, based on the Illumina’s manifest file and USCS genome browser (https://genome.ucsc.edu (accessed on 6 April 2020)). Pathway enrichment analysis of the identified CpGs was conducted using the *gometh* function [42] in the R package to better understand their biological functionality. To help understand the interconnections of genes within each pathway, the Cytoscape bioinformatics webtool (https://cytoscape.org (accessed on 1 November 2021)) was utilized.

## 3. Results

Since our study focused on asthma acquisition starting from the age of 10 years in the IOWBC, subjects with asthma at four years were excluded. Participants in the IOWBC with both asthma transition and DNAm data available at ages 10 and 18 years were included in the study. The subsamples represented the complete IOWBC (such that no asthma existed at an age of 4 years) with respect to asthma acquisition, active and second-hand smoking, and atopy status (Table 1 and Table 2).

Additional characteristics of asthmatics in the IOWBC were assessed using data at 18 years, including overnight hospital stays related to asthma (Supplement Table S1, the number of wheeze attacks in the last 12 months (Supplement Table S2), FeNO (fractional exhaled nitric oxide) levels, and the ratio of the forced expiratory volume in the first one second (FEV_1_) to the forced vital capacity of the lungs (FVC) (Supplement Table S3). The majority of asthmatic patients did not have any overnight hospital stays, and most of them had 1–4 wheeze attacks in the last 12 months (Supplement Tables S1 and S2). Subjects with and without asthma acquisition were compared for FeNO levels and the FEV_1_/FVC ratio. Subjects who acquired asthma had higher FeNO levels and a lower FEV_1_/FVC ratio compared to those who did not acquire asthma in both transition periods (Supplement Table S3).

In total, 55 CpGs for males and 183 CpGs for females in the IOWBC passed screening based on their potential associations with asthma acquisition from 10 to 18 years of age. These CpGs were included in subsequent analyses for their longitudinal associations of DNAm with asthma acquisition from pre- to post-adolescence and from post-adolescence to young adulthood and for interaction effects between DNAm and transition periods, using logistic regressions with repeated measurements. The utilization of logistic regressions with repeated measures allowed us to identify effect sizes with a higher statistical power, even if the number of “cases” (asthma acquisitions) was relatively small.

**Figure 1:**
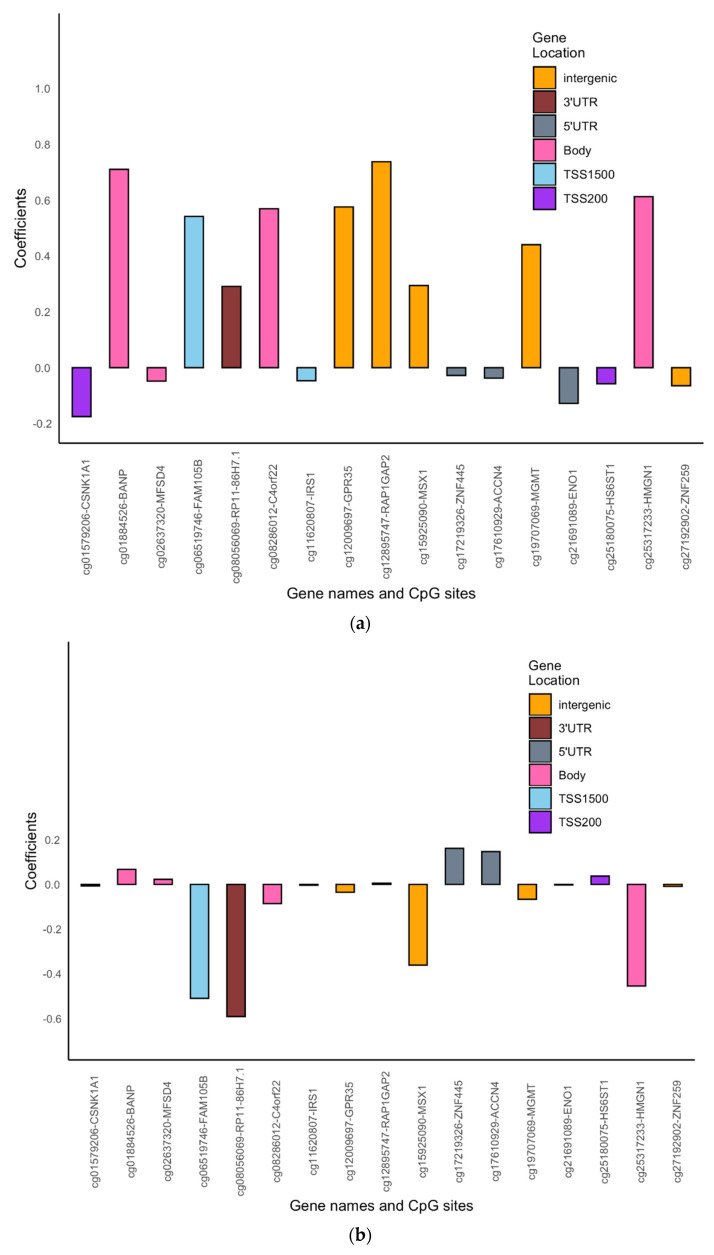
(**a**): Bar graph showing the direction of the DNAm effect at each of the 17 identified CpGs in the IOWBC for pre- to post-adolescence asthma acquisition in males. Gene names corresponding to each CpG site are also labelled on the X-axis. (**b**): Bar graph showing the direction of effect at each of the 17 identified CpGs in the IOWBC for post-adolescence to adulthood asthma acquisition in males. Gene names corresponding to each CpG site are also labelled on the X-axis.

**Figure 2:**
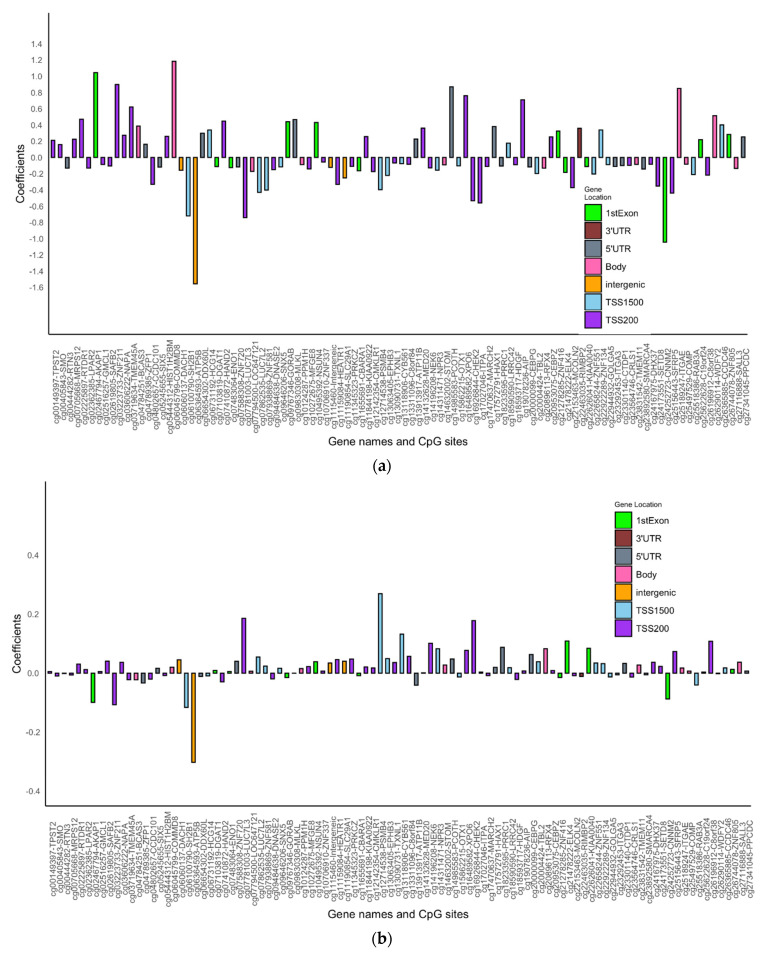
(**a**): Bar graph showing the direction of effect at each of the 98 identified CpGs in the IOWBC for pre- to post-adolescence asthma acquisition in females. Gene names corresponding to each CpG site are also labelled on the X-axis. (**b**): Bar graph showing the direction of effect at each of the 17 identified CpGs in the IOWBC for post-adolescence to adulthood asthma acquisition in females. Gene names corresponding to each CpG site are also labelled on the X-axis.

After adjusting for multiple testing by controlling the FDR to be 0.05, statistically significant interaction effects of DNAm and the transition period were observed at 17 CpGs in males and 98 CpGs in females (no common CpGs were identified between males and females), controlling for atopy status and active and second-hand smoking (Figure 1 and Figure 2, Supplement Table S2). Of the 17 identified CpGs in males, 4 CpGs (23.5%) were located in the promoter region, while for the 98 CpGs identified in females, a much larger portion of CpGs (54 CpGs, 55.1%) were in the promoter region. For 7 of the 17 CpGs in males, an increase in DNAm was associated with increased odds of asthma acquisition in the 10–18-year transition period but decreased odds in the 18–26-year period (Figure 1, Supplement Table S4). For 47 of the 98 CpGs in females, an increase in DNAm was associated with decreased odds of asthma acquisition in the 10–18-year transition period but increased odds in the 18–26-year period (Figure 2, Supplement Table S5). In addition, the overall effect sizes at the first transition period were larger than the effect sizes in the second transition period.

**Figure 3:**
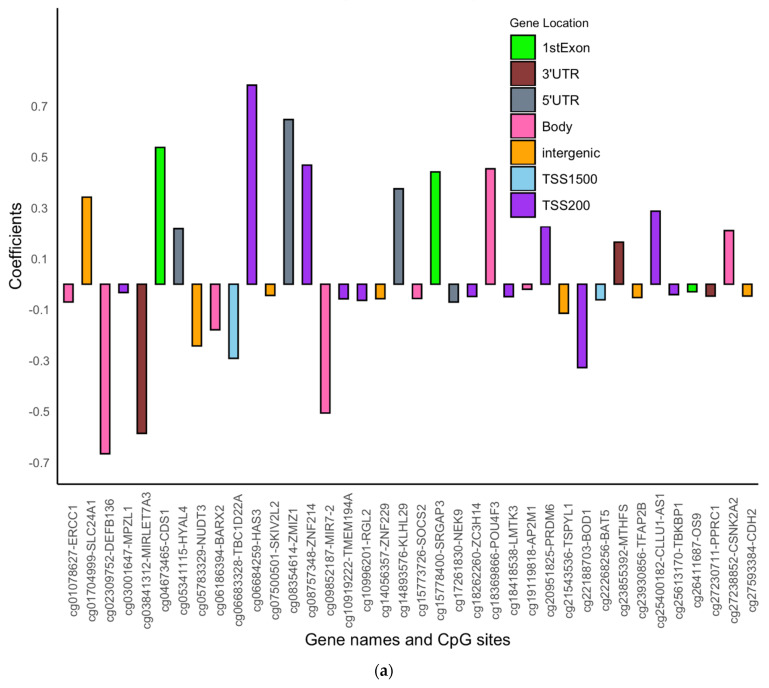

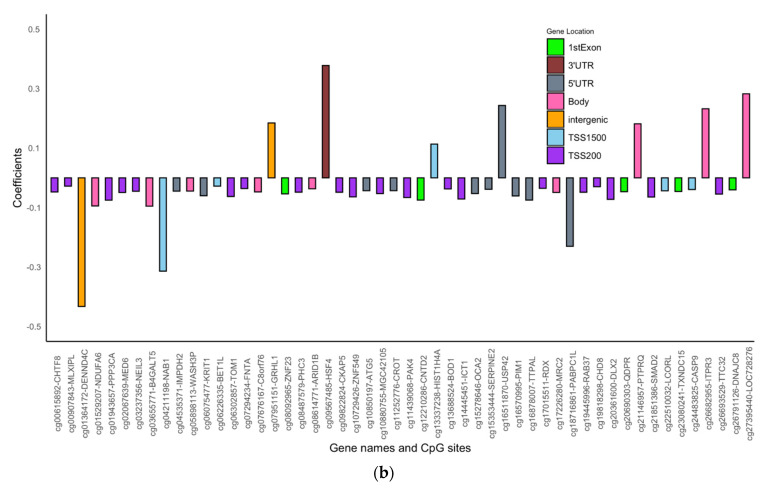
(**a**): Bar graph showing the main effects of DNAm on asthma acquisition at each of the 38 identified CpGs in the IOWBC in males. Gene names corresponding to each CpG site are also labelled on the X-axis. (**b**): Bar graph showing the main effects of DNAm on asthma acquisition at each of the 52 identified CpGs in the IOWBC in females. Gene names corresponding to each CpG site are also labelled on the X-axis.

For CpGs not showing significant interaction effects between DNAm and the transition period, we assessed the main effects of DNAm on asthma acquisition via logistic regression models with repeated measures. After adjusting for multiple testing at FDR = 0.05, we identified 38 CpGs in males and 52 CpGs in females (Figure 3, Supplement Table S3) showing an association of DNAm with asthma acquisition status (with no common CpGs between males and females). Of the 38 CpGs in males, 13 CpGs (34.2%) were in the promoter region, while for the 52 CpGs identified in females, a much larger portion of CpGs (25 CpGs, 48.1%) were in the promoter region. At 25 of the 38 CpGs in males, an increase in DNAm was associated with decreased odds of asthma acquisition (Supplement Table S6). At 45 of the 52 CpGs in females, an increase in DNAm was associated with decreased odds of asthma acquisition (Supplement Table S7). Overall, the effect sizes of DNAm on asthma acquisition were larger in males than in females.

**Figure 4:**
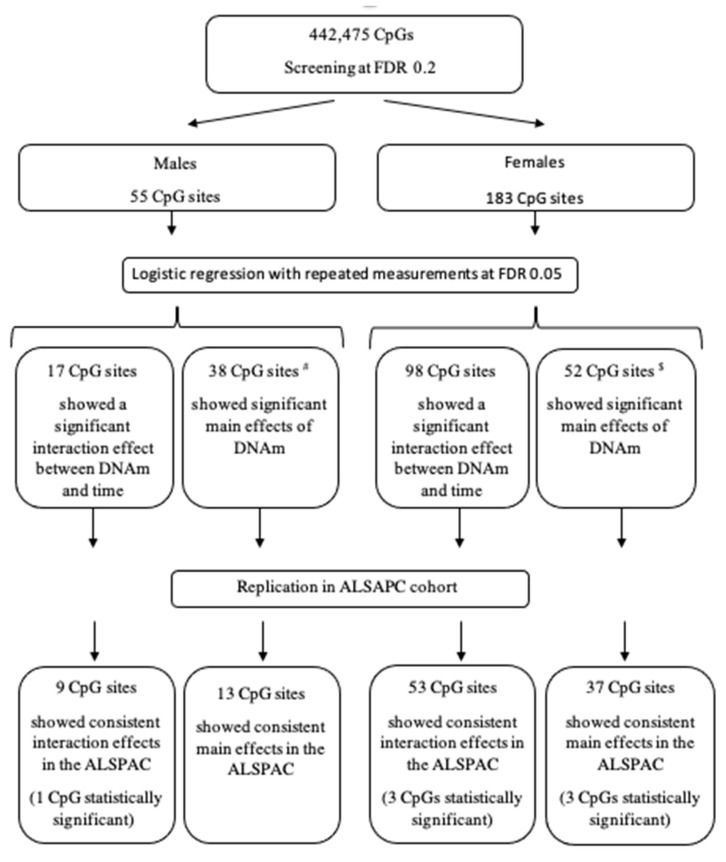
Flowchart of the study design and brief summaries of findings in each step. ^#^—Out of 38 CpGs after excluding significant interactions. ^$^–Out of 85 CpGs after excluding significant interactions.

Altogether, we identified 115 CpGs (17 in males) showing interactions with the transition period and 90 CpGs (38 in males) showing main effects (excluding CpGs with interaction effects), leading to a total of 205 identified CpGs. We further tested these CpGs in the ALSPAC cohort. For the 115 CpGs (17 in males) showing interaction effects in the IOWBC, consistent directions of interaction effects were observed at 9 CpGs in males, with 1 CpG being statistically significant, and 53 CpGs in females, with 3 CpGs being statistically significant, compared to the directions of associations identified in the IOWBC (Supplement Tables S4 and S5). Of the 9 CpGs showing consistent interactions in males, 2 CpGs (22.2%) were in the body region of the gene, while of the 53 such CpGs in females, 34 (64.2%) were in the promoter region. For the 90 CpGs (38 in males) showing main effects on asthma acquisition, 13 CpGs in males (3 CpGs (23.1%) in the promoter region) and 37 CpGs in females (19 CpGs (51.4%) in the promoter region) showed consistent directions of main effects (Supplement Tables S6 and S7) between the two cohorts. The flowchart of the study along with brief summaries of results are shown in Figure 4.

Furthermore, at 112 CpGs (with interaction or main effects) showing consistent associations between the two cohorts, we evaluated the association of DNAm with FeNO and FEV_1_/FVC. The majority of the findings for FeNO and FEV_1_/FVC were consistent with our findings, although statistical significance was not reached, potentially due to the large variations in these two lung function measurements. More specifically, a higher DNAm was associated with higher odds of asthma acquisition, higher FeNO levels, and a lower FEV_1_/FVC measurement, and vice versa (Supplement Tables S8–S11).

Pathway enrichment analyses were conducted based on the IOWBC-discovered CpGs for each sex (55 in males and 150 in females, with 205 CpGs in total) to better understand their biological functionality. These CpGs were mapped to 54 and 149 genes in males and females, respectively. Using these CpGs in the *gometh* function in R, we identified 212 biological processes in males and 228 in females that were enriched at a *p*-value of 0.05. Although none of the biological processes survived multiple testing at an FDR of 0.05, genes involved in the top processes for each sex based on statistical significance (top 10 processes in Table 3 and Table 4, Supplement Figure S1 for the network among these genes) were potentially important and may deserve a further assessment. For males, multiple biological processes among the top 10 for males focused on catabolic processes (breakdown of glucose for energy), while for females, they were biosynthetic processes (synthesizing glucose from food). Among these top processes identified for males, 13 genes corresponding to the identified CpGs were involved in those processes, and for females, 60 genes were involved (Supplement Tables S12 and S13). Of the 13 genes, the CpGs on five (~39%) genes showed consistent associations (interaction or main effects) between the IOWBC and ALSPAC, and of the 60 genes, 34 (~57%) genes showed such consistency between the two cohorts.

For DMR enrichment analysis, we used CpGs in the screening process that were statistically significant at an FDR of 0.4 to cover epigenetic information comprehensively on asthma acquisition. In total, 427 CpGs in males and 372 CpGs in females were included in the analysis. We identified three DMRs in males and three DMRs in females (Table 5).

## 4. Discussion

We assessed the longitudinal association of DNAm measured at earlier ages with asthma acquisition at later ages for each sex based on data from two independent cohorts, with the IOWBC as the discovery cohort and the ALSPAC as the replication cohort. In the IOWBC, at 205 CpGs, pre-adolescence DNAm was shown to be associated with the odds of asthma acquisition from pre- to post-adolescence, and post-adolescence DNAm was associated with asthma acquisition from post-adolescence to adulthood. At 112 of these 205 CpGs (54.6%), consistent associations were observed in the ALSPAC cohort, including statistically significant findings at seven CpGs. These 112 CpGs included 62 CpGs (nine in males) showing transition-specific associations with asthma acquisition, in that the association of DNAm with asthma acquisition at these 62 CpGs was different between the pre- to post-adolescence transition period and the post-adolescence to adulthood transition period.

Our findings also indicated significant differences between males and females. For the 62 CpGs showing consistent transition-specific effects between the two cohorts, at most of the CpGs in males, we found that an increase in DNAm was associated with increased odds of asthma acquisition during the period from pre- to post-adolescence transition, while for the next transition period, at most of the CpGs, increased DNAm was associated with decreased odds. However, in females, at most of the CpGs, the associations were opposite compared to those in males; in females, an increase in DNAm was shown to be associated with decreased odds of asthma acquisition from pre- to post-adolescence at most CpGs but with increased odds at most of the CpGs in the transition period from post-adolescence to adulthood. Among the 50 CpGs (13 in males) showing main effects on asthma acquisition, at most of these CpGs, an increase in DNAm was associated with a decreased odds of asthma acquisition for both males and females, but the proportion of such CpGs was larger in females than in males. Furthermore, the effect sizes were overall weaker in females than in males. Before adolescence, asthma was more prevalent in males, but during adolescence, more females acquired asthma, and the prevalence of asthma in females surpassed that of males. The inconsistent patterns of association of DNAm with the odds of asthma acquisition between males and females seemed to be related to the gender-reversal phenomenon of asthma prevalence from pre- to post-adolescence. Although there was a possibility of false positive findings, the complete non-overlapping of CpGs between males and females seems to suggest that the underlying epigenetic mechanisms of asthma acquisition may be different for each sex. Another possibility is the existence of unique DNAm markers in each sex being involved in asthma acquisition. We speculate that these differences (either in mechanisms or markers) may help explain the observed gender reversal phenomenon in asthma prevalence [43].

Although we did not identify statistically significant biological processes after adjusting for multiple testing, biological processes involved in host immune function related to IL-7 (i.e., the interleukin-7-mediated signalling pathway, responses to interleukin-7, and cellular responses to interleukin-7) were among the top processes determined based on statistical significance. These processes, along with the genes involved, may lead to potential cellular targets for prevention, precise diagnosis, and treatment [44,45,46]. The *IRS1* gene was involved in the processes related to IL-7, and its mapped CpG (cg11620807) showed a consistent association between the two cohorts. IL-7 signalling has been suggested to promote the immunopathogenesis of asthma [47,48], indicating the potential informativity of the identified CpGs on asthma acquisition. The identification of cell metabolism-related biological processes helps us to better understand the underlying pathogenesis of asthma acquisition. Cellular metabolism consisting of interconnected catabolic and anabolic pathways plays an important role in immunity and the inflammatory response [49]. Defective innate immune responses in asthma are driven by abnormal metabolic programming [49]. A recent study has shown the importance of metabolism for immune homeostasis in allergic diseases, including asthma. The metabolic status of T cells and macrophages has been shown to be associated with the phenotypes of allergic inflammation [50]. For example, Rodriguez-Perez et al. demonstrated altered fatty acid metabolism and reduced stearoyl-coenzyme a desaturase (SCD) activity in asthma compared to controls [51]. Similarly, Kuo et al. highlighted the significance of metabolic characterization of immune cells to identify asthma endotypes [52]. In addition, the *TMEM194A* gene, identified based on DMR analyses in males, has previously been shown to be associated with asthma in the GWAS catalogue [53]. For females, gene *SERPINE2* in one of the identified DMRs has been connected with asthma based on genetic studies [54].

The gene *AKAP1*, mapped to cg02467794, showing consistent and statistically significant interaction effects in both cohorts in females, has been shown to be associated with asthma in the Agricultural Lung Health Study [53]. Although there was no overlap in the identified CpGs between males and females, the gene *ENO1* was among the mapped genes of the IOWBC-discovered CpGs in both sexes. The detection of IgG autoantibodies to alpha-enolase has been shown to be the most significant indicator for distinguishing severe asthma from mild-to-moderate asthma (OR = 5.2, 95% CI = 2.1–12.9, *p*-value <0.001). It has been shown that alpha-enolase, an autoantigen, is associated with severe asthma [55]. The connection of gene *ENO1* with asthma acquisition shown in our study is consistent with its differentiation between severe and mild-to-moderate asthma. Further assessment of CpGs located on this gene is likely to benefit the potential of the CpGs as epigenetic markers for asthma acquisition.

The strength of this study exists in its focus on the longitudinal assessment of asthma acquisition at two important transition periods, pre- to post-adolescence and to young adulthood, along with DNAm at two critical time points, pre- and post-adolescence. In a candidate gene approach, we have previously demonstrated the concurrent association of DNAm changes at specific CpGs in genes of the Th2 pathway and asthma transition from pre- to post-adolescence in females [25] and the sex-specific association of DNAm changes with asthma acquisition during this transition period [8]. Both of these studies were based on concurrent modelling, i.e., DNAm changes and asthma acquisition were assessed in the same transition period. However, in the current study, the focus was on DNAm at earlier ages with asthma acquisition at later ages, and two acquisition periods were examined in the study (pre- to post-adolescence and post-adolescence to adulthood). Our earlier studies also examined the longitudinal association of DNAm with lung function measurements and their trajectories [56,57,58]. In this study, our focus was on asthma acquisition, while lung function represented a feature of asthma. To our knowledge, this is the first longitudinal study to assess the epigenetics of asthma acquisition from pre- to post-adolescence and post-adolescence to young adulthood with respect to gender and transition-period-specificity.

Although for the CpGs discovered in IOWBC, more than 50% showed consistent findings in the ALSPAC, statistical significance was observed at a small number of CpG sites. One reason for this lack of significance might be the age differences between the two cohorts. In addition, there was a potential concern of data double dipping. However, we do not see this as a significant concern in that the statistical model applied in the screening process (linear regression without covariates such as being atopic and smoking status) was different from the model in the final analyses (logistic regression with potential covariates). We also noticed that the number of asthma acquisitions at each age was relatively small. However, our utilization of repeated measures in the regression analyses had a potential to ease this concern, and the inclusion of the ALSPAC replication cohort to further examine the IOWBC findings with a focus on a consistent direction of associations further relieved our worries to a certain extent. Another potential limitation was in the design of data analyses, which focused on each individual CpG site. However, CpG sites might be correlated and work jointly to impact the risk of asthma acquisition, which certainly deserves future investigations accompanied by carefully designed analytical plans. Finally, both cohorts, although independent, were mainly Caucasians. Thus, our findings are likely limited to only this population. Nevertheless, the identified CpGs based on two independent cohorts have a potential to guide future studies on asthma acquisition prediction at different transition periods.

## Figures and Tables

**Table 1:** Asthma acquisition and never asthma subjects included in the current study compared to subjects in the complete cohort for the 10–18-year period (IOWBC).

Variables N (%)		Females			Males		
		Subsample N = 102; n (%)	Complete Cohort N = 431; n (%)	*p*-Value	Subsample N = 133; n (%)	Complete Cohort N = 402; n (%)	*p*-Value
Asthma transition	Acquisition	7 (6.86)	41 (9.51)	0.40	11 (8.27)	26 (6.47)	0.98
	Never Asthma	95 (93.14)	390 (90.49)		122 (91.73)	376 (93.53)	
Active smoking	Yes	25 (23.36)	120 (25.86)	0.59	33 (21.85)	105 (22.98)	0.48
	No	82 (76.64)	344 (74.14)		118 (78.15)	352 (77.02)	
Second-hand smoking	Yes	54 (50)	212 (44.92)	0.34	66 (43.42)	204 (43.59)	0.78
	No	54 (50)	260 (55.08)		86 (56.58)	264 (56.41)	
Atopy	Yes	14 (12.96)	76 (19.1)	0.14	42 (27.81)	108 (27.91)	0.97
	No	94 (87.04)	322 (80.9)		109 (72.19)	279 (72.09)	

**Table 2:** Asthma acquisition and never asthma subjects included in the current study compared to subjects in the complete cohort for the 18–26-year period (IOWBC).

Variables N (%)		Females			Males		
		Subsample N = 156; n (%)	Complete Cohort N = 330; n (%)	*p*-Value	Subsample N = 121; n (%)	Complete Cohort N = 286; n (%)	*p*-Value
Asthma transition	Acquisition	5 (3.21)	12 (3.64)	0.81	3 (2.48)	10 (3.5)	0.59
	Never Asthma	151 (96.79)	318 (96.36)		118 (97.52)	276 (96.5)	
Active smoking	Yes	46 (27.54)	107 (26.95)	0.89	46 (31.94)	99 (28.95)	0.51
	No	121 (72.46)	290 (73.05)		98 (68.06)	243 (71.05)	
Second-hand smoking	Yes	35 (20.96)	98 (24.75)	0.33	27 (18.75)	80 (23.39)	0.26
	No	132 (79.04)	298 (75.25)		117 (81.25)	262 (76.61)	
Atopy	Yes	55 (29.73)	115 (32.63)	0.36	73 (42.2)	146 (46.65)	0.35
	No	130(70.27)	227 (66.37)		100 (57.8)	167 (53.35)	

**Table 3:** The top 10 most statistically significant GO terms and their biological processes from pathway enrichment analysis along with the number of genes in each pathway for males, for the identified CpGs.

GO term	Biological Processes	*p*-Value	No. of Genes
GO:1901575	organic substance catabolic process	0.0005	12
GO:0046657	folic acid catabolic process	0.001	1
GO:0042219	cellular modified amino acid catabolic process	0.002	1
GO:0038111	interleukin-7-mediated signaling pathway	0.002	2
GO:1990261	pre-mRNA catabolic process	0.002	1
GO:0071544	diphosphoinositol polyphosphate catabolic process	0.002	1
GO:0042365	water-soluble vitamin catabolic process	0.003	1
GO:0009056	catabolic process	0.003	13
GO:0098760	response to interleukin-7	0.003	2
GO:0098761	cellular response to interleukin-7	0.003	2

**Table 4:** The top 10 most statistically significant GO terms and their biological processes from pathway enrichment analysis along with the number of genes in each pathway for females, for the identified CpGs.

GO term	Biological Processes	*p*-Value	No. of Genes
GO:0019438	aromatic compound biosynthetic process	0.0004	57
GO:0032774	RNA biosynthetic process	0.0007	49
GO:0008589	regulation of smoothened signaling pathway	0.0007	5
GO:0018130	heterocycle biosynthetic process	0.0008	56
GO:1903506	regulation of nucleic acid-templated transcription	0.0008	49
GO:2001141	regulation of RNA biosynthetic process	0.0008	49
GO:1901362	organic cyclic compound biosynthetic process	0.001	57
GO:0034654	nucleobase-containing compound biosynthetic process	0.001	55
GO:0006355	regulation of transcription, DNA-templated	0.001	48
GO:0009757	hexose-mediated signaling	0.001	2

**Table 5:** Differentially methylated regions (DMRs) for asthma acquisition identified by the *DMRcate* package for males and females.

Sex	Chr. ^$^	Start ^#^	End ^&^	Gene ^£^	CpGs ^¥^	*p*-Value
M	3	196065106	196065569	*TM4SF19*	cg05556202, cg05445326	4.89 × 10^−206^
	12	57472396	57472611	*TMEM194A*	cg10919222, cg09934365	3.03 × 10^−150^
	17	27899874	27899966	*TP53I13*	cg05877788, cg04498198	2.69 × 10^−53^
F	2	224903369	224903487	*SERPINE2*	cg15353444, cg11719885	5.84 × 10^−177^
	1	156721844	156722068	*HDGF*	cg04402095, cg18593717	5.35 × 10^−162^
	17	79633496	79633565	*CCDC137, C17orf90*	cg199963747, cg11820993	1.70 × 10^−160^

^$^ Chr.: Chromosome; ^#^ Start: Start position of the region; ^&^ End: End position of the region; ^£^ Gene: Genes corresponding to the CpGs in the region; ^¥^ CpGs: CpGs in the region.

## Data Availability

The datasets used and/or analysed during the current study are available from the corresponding author on reasonable request. For the ALSPAC data, please contact the ALSPAC executive committee (alspac-exec@bristol.ac.uk (accessed on 12 August 2019)).

## Supplementary Materials

The following supporting information can be downloaded at: https://www.mdpi.com/article/10.3390/jpm12020202/s1. Supplement Table S1. Distribution of hospital overnight stays in males and females with asthma at an age of 18 years. Supplement Table S2. Comparison of the number of wheeze attacks in last 12 months amongst males and females with asthma at an age of 18 years. Supplement Table S3. Comparison of subjects with and without asthma acquisition for transition period 1 from pre- to post-adolescence. Supplement Table S4. Association of DNAm with asthma acquisition from pre- to post-adolescence and post-adolescence to young adulthood at 17 CpGs in males that are transition-period-specific. The significant interaction between DNAm and the transition period on asthma acquisition identified in the IoW(a) cohort was further tested in the ALSPAC(b) cohort. The 10–18-year transition period was the reference group. Supplement Table S5. Association of DNAm with asthma acquisition from pre- to post-adolescence and post-adolescence to young adulthood at 98 CpGs in females that are transition-period-specific. The significant interaction between DNAm and the transition period on asthma acquisition identified in the IoW(a) cohort was further tested in the ALSPAC(b) cohort. The 10–18-year transition period was the reference group. Supplement Table S6. Association of DNAm with asthma acquisition from pre- to post-adolescence and post-adolescence to young adulthood at 38 CpGs in males that are transition-period-non-specific. The significant effects of DNAm on asthma acquisition identified in the IoW(a) cohort were further tested in the ALSPAC(b) cohort. Supplement Table S7. Association of DNAm with asthma acquisition from pre- to post-adolescence and post-adolescence to young adulthood at 53 CpGs in females that are transition-period-non-specific. The significant effects of DNAm on asthma acquisition identified in the IoW(a) cohort were further tested in the ALSPAC(b) cohort. Supplement Table S8. Results of the longitudinal association of DNAm (ages 10 and 18 years) with asthma acquisition (AA) (10–18 and 18 –26 years), FeNO (ages 18 and 26 years), and FEV_1_/FVC (ages 18 and 26 years) at the 9 identified CpGs in males showing interaction effects. Supplement Table S9. Results of the longitudinal association of DNAm (ages 10 and 18 years) with asthma acquisition (AA) (10–18 and 18 –26 years), FeNO (ages 18 and 26 years), and FEV_1_/FVC (ages 18 and 26 years) at the 13 identified CpGs in males showing main effects. Supplement Table S10. Results of the longitudinal association of DNAm (ages 10 and 18 years) with asthma acquisition (AA) (10–18 and 18–26 years), FeNO (ages 18 and 26 years), and FEV_1_/FVC (ages 18 and 26 years) at the 53 identified CpGs in females showing interaction effects. Supplement Table S11. Results of the longitudinal association of DNAm (ages 10 and 18 years) with asthma acquisition (AA) (10–18 and 18–26 years), FeNO (ages 18 and 26 years), and FEV_1_/FVC (ages 18 and 26 years) at the 37 identified CpGs in females showing main effects. Supplement Table S12. The top 10 most statistically significant GO terms and their biological processes from pathway enrichment analyses along with their genes’ names in each pathway for males, for the identified CpGs. Supplement Table S13. The top 10 most statistically significant GO terms and their biological processes from pathway enrichment analyses along with their genes’ names in each pathway for females, for the identified CpGs. Supplement Figure S1A: Graphical visualization using Cytoscape for the top 10 statistically significant pathways and genes included in these pathways for males. Gray oval: pathways; blue rectangles: genes. Supplement Figure S1B: Graphical visualization using Cytoscape for the top 10 statistically significant pathways and genes included in these pathways for females. Gray oval: pathways; blue rectangles: genes.

## Declarations

Ethics approval and consent to participate. The Isle of Wight Local Research Ethics Committee approved the 10-years assessment as well as the collection of blood for genetic studies into asthma and allergy (No. 18/98, dated 07/20/1998). For the 18-year follow-up, ethical approval was given by the Isle of Wight, Portsmouth, and SE Hampshire Local Research Ethics Committee (No. 06/Q1701/34, dated 06/16/2006). The Internal Review Board (IRB) at the University of Memphis (FWA00006815, IRB ID: #3917) gave approval for data analysis at the University of Memphis. Written informed consent was obtained from parents during in-person visits. For participants assessed by phone interview, consent was documented on the consent form with the name of the person giving consent, and the name and signature of the person taking the form were recorded. For ALSPAC, ethical approval for the study was obtained from the ALSPAC Ethics and Law Committee and the Local Research Ethics Committees. Consent for collection of biological samples was provided in accordance with the Human Tissue Act (2004). Informed consent for the use of data collected via questionnaires and clinics was obtained from participants, following the recommendations of the ALSPAC Ethics and Law Committee at the time. For the age of seven years, the United Bristol Healthcare Trust: E4168 (ALSPAC Hands on Assessments at Age Seven), Southmead Health Services: 67/98 (Avon Longitudinal Study of Pregnancy and Childhood (ALSPAC)—Hands on Assessments at Age Seven), and the Frenchay Healthcare Trust: 98/52 (Avon Longitudinal Study of Pregnancy and Childhood (ALSPAC) provided approval. For the age of 15 years, the Central and South Bristol Research Ethics Committee (UBHT): 06/Q2006/53 Avon Longitudinal Study of Parents and Children (ALSPAC) Hands on Assessments: Teen Focus 3 (Focus 15+) were used, and for the age of 17 years, North Somerset and South Bristol Research Ethics Committee: 08/H0106/9 Avon Longitudinal Study of Parents and Children (ALSPAC) Hands on Assessments: Teen Focus 4 (Focus 17+) were used. Full details of ethical approvals (local committees and approval numbers) are available at http://www.bristol.ac.uk/media-library/sites/alspac/documents/governance/Research%20Ethics%20Committee%20approval%20references.pdf (accessed on 12 August 2019).

## Abbreviations

CpG	5′-C-Phosphate-G-3′
DNAm	Deoxyribonucleic acid methylation
DMRs	Differentially Methylated Regions
FDR	False Discovery Rate
