# Supplementary material for: DNA Methylation and Asthma Acquisition during Adolescence and Post-Adolescence, an Epigenome-Wide Longitudinal Study
Source: J Pers Med. 2022 Feb 2;12(2):202. doi: 10.3390/jpm12020202 (PMC8877984; doi:10.3390/jpm12020202)
Supplement: Supplementary file 1 [file jpm-12-00202-s001.zip › jpm-1558366-supplementary figure.pdf]

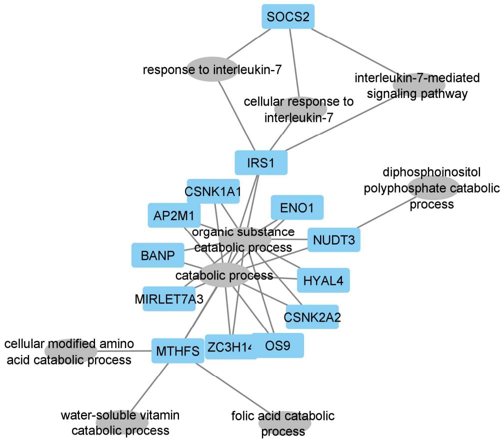

(**A**)

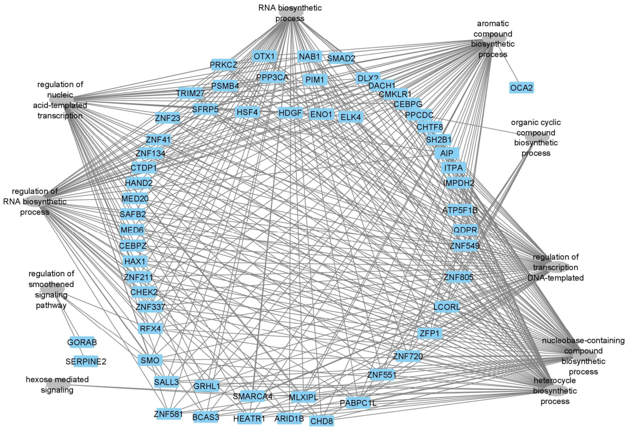

(**B**)

Figure S1. (**A**) Graphical visualization using Cytoscape for the top 10 statistically significant pathways and genes included in these pathways for males. Gray oval: pathways; blue rectangles: genes. (**B**) Graphical visualization using Cytoscape for the top 10 statistically significant pathways and genes included in these pathways for females. Gray oval: pathways; blue rectangles: genes.
